# Feasibility and effect of life skills building education and multiple micronutrient supplements versus the standard of care on anemia among non-pregnant adolescent and young Pakistani women (15–24 years): a prospective, population-based cluster-randomized trial

**DOI:** 10.1186/s12978-018-0547-y

**Published:** 2018-05-30

**Authors:** Jo-Anna B. Baxter, Yaqub Wasan, Sajid B. Soofi, Zamir Suhag, Zulfiqar A. Bhutta

**Affiliations:** 1 Centre for Global Child Health, The Hospital for Sick Children, Toronto, ON M6S 1S6 Canada; 2 Department of Nutritional Sciences, University of Toronto, Toronto, ON Canada; 3 Centre of Excellence in Women and Child Health, Aga Khan University, Stadium Road, Karachi, 74800 Pakistan

**Keywords:** Adolescence, Young adult, Nutrition, Micronutrients, Education, Preconception, Anemia, Empowerment

## Abstract

**Background:**

Adolescence is a critical period for physical and psychological growth and development, and vitamin and mineral requirements are correspondingly increased. Health and health behaviours correspond strongly from adolescence to adulthood. Developing a preconception care package for adolescent and young women in resource-limited settings could serve to empower them to make informed decisions about their nutrition, health, and well-being, as well as function as a platform for the delivery of basic nutrition-related interventions to address undernutrition.

**Methods:**

In this population-based two-arm, cluster-randomized, controlled trial of life skills building education (provided bi-monthly) and multiple micronutrient supplementation (provided twice-weekly; UNIMMAP composition), we aim to evaluate the effectiveness of the intervention on the prevention of anemia (hemoglobin concentration < 12 g/dL) among adolescent and young women (15–24 years) in Matiari district, Pakistan compared to the standard of care. Several secondary objectives related to nutrition (anthropometry [height, weight, middle upper arm circumference (MUAC)], nutritional status [iron, vitamin A, vitamin D]); general health (morbidity, mortality); and empowerment (age at marriage, completion of the 10th grade, use of personal hygienic materials during menstruation) will also be assessed. Participants will be enrolled in the study for a maximum of 2 years.

**Discussion:**

Empowering adolescent and young women with the appropriate knowledge to make informed and healthy decisions will be key to sustained behavioural change throughout the life-course. Although multiple micronutrient deficiencies are known to exist among adolescent and young women in low-resource settings, recommendations on preconception multiple micronutrient supplementation do not exist at this time. This study is expected to offer insight into providing an intervention that includes both education and supplements to non-pregnant adolescent and young women for a prolonged duration of time within the existing public health programmatic context.

**Trial registration:**

This study is part of the Matiari emPowerment and Preconception Supplementation (MaPPS) Trial. The MaPPS Trial was registered retrospectively on clinicaltrials.gov (Identifier: NCT03287882) on September 19, 2017.

**Electronic supplementary material:**

The online version of this article (10.1186/s12978-018-0547-y) contains supplementary material, which is available to authorized users.

## Plain English summary

In low- and middle-income countries many adolescent and young women do not consume enough healthy and nutrient-rich foods to meet their daily needs. Data show that adolescent and young women in these places are also more likely to become pregnant. If a woman is undernourished when she becomes pregnant, it can have a negative effect on her health, pregnancy, and infant. One way to improve adolescent and young women’s health before they become pregnant is to provide a program that aims to help them make healthy decisions and improve their nutrition. This is often called preconception care. In this study in rural Pakistan, we have designed a culturally-appropriate preconception care package that includes life skills building education, to help study participants make better decisions about their health and educate them about their rights, and multiple micronutrient supplements, since they have limited access to foods with the micronutrients that they need. Study participants (15–24 years) are enrolled for 2 years, and we will assess several factors about their nutrition, health, and well-being over this time. The main effect we aim to determine is how many participants have anemia among those who receive the preconception care package compared to those who do not receive it. Ultimately, we hope that what we learn in this study will lead us to make recommendations about policies around preconception care for adolescent and young women so that they can live healthier lives and make healthier decisions.

## Background

Within the Sustainable Development Goals, promoting strategies to ensure the survival, health, and well-being of adolescent girls, women, and their offspring has been recognized as an essential component to ending poverty [1]. Adolescence, in particular, is considered a neglected yet critical period for physical and psychological growth and development, given the transition from childhood to reproductive adulthood [2]. Pregnancies to adolescents are associated with a higher risk of complications and maternal and child mortality [3], and contribute to 23% of the burden of disease arising from pregnancy and childbirth [4]. When an adolescent becomes pregnant in a resource-limited setting, her access to education generally stops as a result; yet, educating girls is very effective in raising overall economic productivity, lowering infant and maternal mortality, improving nutrition, and promoting health [5]. Collectively, this makes determining interventions to improve health outcomes from adolescence of public health importance [2].

Nutritional deficiencies are risk factors known to exist from adolescence, and have important consequences for future maternal and newborn health. During adolescence, vitamin and mineral requirements are incrementally increased to support one’s ongoing physical development and the nutritional strain of menstruation [6]. Given the limited intake of animal products, fruits, vegetables, and fortified foods in low- and middle-income countries, undernutrition can be widely prevalent [7]. Deficiencies in multiple micronutrients (MMN) are of particular concern given their direct effects, such as iron deficiency anemia and iodine deficiency disorders, and their indirect effects, like increased risk of serious infectious disease [6]. The burden of undernutrition and its downstream consequences could be prevented throughout the life course by using appropriate nutrition-specific and nutrition-sensitive interventions, such as MMN supplementation and the empowerment of women to make informed decisions, respectively [5].

Since health and health behaviours correspond strongly from adolescence to adulthood, habits acquired during adolescence could have significant implications later in life [2]. As such, developing a preconception care package for adolescent and young women could serve to empower them to make informed decisions about their health and well-being [8], and function as a platform for the delivery of basic nutrition-related interventions to address undernutrition [9]. Incorporating a life skills building education (LSBE) element will be important to sustainable and proficient uptake in improving their own general health, pregnancy outcomes, and future health of their children [8]. However, at this time there is limited understanding as to what health information should be provided and how within preconception care.

We hypothesize that the programmatic provision of LSBE and MMN supplements to non-pregnant adolescent and young women 15–24 years of age in a population-based setting in rural Pakistan will improve selected nutrition, health, and empowerment-related outcomes compared to those who receive the standard of care. For those who receive the intervention, we expect that there will be a decrease in the prevalence of anemia. Conducting this trial within the context of the existing public health setting will allow us to assess the *effectiveness* of the intervention, and offer important insight around how to achieve coverage in the trial setting.

## Methods

The Matiari emPowerment and Preconception Supplementation (MaPPS) Trial is a two-arm, cluster-randomized, controlled trial of LSBE and MMN supplementation provided in a programmatic context to evaluate the impact on pre-identified nutrition and health outcomes among adolescent and young women, and their infants born within the context of the trial. Depending on whether participants become pregnant or not, there are three phases: preconception (maximum 24 months in duration); pregnancy (approximately 9 months in duration); and postpartum (maximum 12 months in duration). A detailed trial protocol has been developed in accordance with the SPIRIT guidelines [10] in a collaboration between the Aga Khan University (AKU; Karachi, Pakistan) and the Hospital for Sick Children (Toronto, Ontario, Canada). This paper addresses the methods for ongoing data collection during the preconception phase of the trial among participants who do not become pregnant, with the methods for the parent trial detailing the pregnancy and postpartum phases appearing elsewhere ([11] unpublished).

The first participant was enrolled on 30 June 2017; enrollment is expected to be ongoing until July 2018; and planned data collection will continue until 2021.

### Objectives

The primary aim of the preconception phase of the MaPPS Trial is to evaluate the impact of LSBE (provided bi-monthly) and supplementation with MMN (provided twice-weekly) versus the standard of care (non-regulated community-based health sessions and no supplements) on anemia (hemoglobin concentration < 12 g/dL) after 2 years among non-pregnant adolescents 15–18.9 years of age at enrolment. There are several secondary objectives to further determine the effect of LSBE and MMN supplementation compared to the standard of care. These relate to nutrition (anthropometry [height, weight, middle upper arm circumference (MUAC)], nutritional status [iron, vitamin A, vitamin D]); general health (e.g., morbidity, mortality); and empowerment (age at marriage, completion of the 10th grade, and use of personal hygienic materials during menstruation).

### Setting and participants

This trial will be conducted in rural settings within Matiari district in Sindh province, Pakistan. Briefly, the nutritional status of adolescent girls in Pakistan is poor, with 51 and 20% experiencing anemia and iron deficiency anemia, respectively [12]. An urban/rural divide is suggested to impact adolescent nutritional status [13], with adolescents living in rural settings having particularly limited dietary diversity [14]. The intervention is situated within Pakistan’s existing Lady Health Worker (LHW) Programme. Each LHW is affiliated with a health facility and visit the homes in her catchment area monthly to carry out community support sessions and disseminate health education messages [15]. LHW-led community sessions have the potential to serve as an important communication channel for adolescents, especially those out of school. However, the content of existing LHW educational materials primarily focuses on priority groups, including children < 5 years, pregnant women, and couples eligible for family planning [16]. Although the LHW curriculum includes some material on topics important to the health of adolescent girls (e.g., dietary practices, anemia), these materials are not widely implemented and not included in community sessions regularly. More detail about the trial setting is described in the parent trial protocol publication ([11] unpublished).

### Eligibility criteria

As the MaPPS Trial is a population-based effectiveness study of LSBE and MMN supplementation from preconception, broad eligibility criteria for participation in the preconception phase were established. These include that the minimum age at enrolment is 15 years and the maximum age is 23 years; adolescent and young women must also report to be physically able to comply with the trial intervention. Adolescent and young women are not eligible to enrol if they are pregnant, participating in a different nutrition trial, or intend to leave the study area. They can be of any marital status. If a participant becomes pregnant, she proceeds to the pregnancy phase of the MaPPS Trial, as detailed in the parent study publication ([11] unpublished).

The micronutrient status and dietary intake of a sub-sample of adolescent participants 15–18.9 years of age at enrolment will also be monitored within the MaPPS Trial. Participants recruited to the dietary assessment subgroup must also be a part of the micronutrient status subgroup.

### Design and sample size

**Fig. 1:**
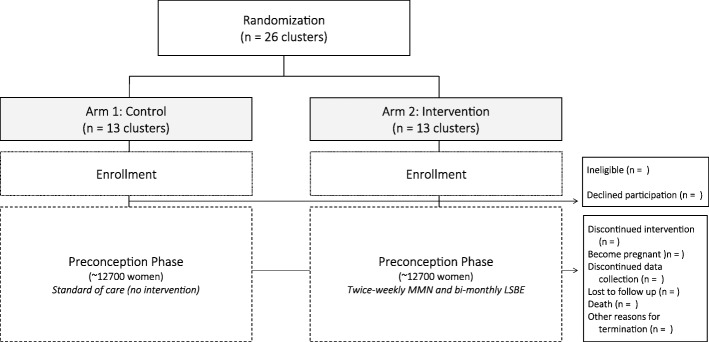
Matiari emPowerment and Preconception Supplementation (MaPPS) Trial flow diagram for participants who do not become pregnant

The MaPPS Trial is a two-arm, parallel, prospective, cluster-randomized, controlled trial (Fig. 1). A cluster-randomized design was chosen to prevent contamination between the control and intervention arms through supplement sharing. The unit of randomization is previously defined and mapped health facility clusters. A total of 26 clusters are available to be randomized (i.e., 13 clusters per arm), and more detail around randomization and allocation concealment are found with the description of the parent study (Baxter et al., unpublished). PASS 11 Software (NCSS, LLC., Kaysville, UT, USA) was used to determine all sample size calculations.

To observe a 20% relative reduction in the prevalence of anemia among non-pregnant adolescent participants 15–18.9 years at enrolment, a 50% baseline prevalence of anemia [12] was assumed. With icc = 0.0256, k = 0.16, accounting for 15% attrition, and probability of type I and type II errors of 0.05 and 0.20, respectively, the number anemic adolescents required per cluster was 66. This equates to 858 anemic adolescents per arm. To achieve this reduction in anemia, 1716 adolescents per arm will be required (3432 total). Given the number of adolescent and young women required to be powered to see a reduction in the primary outcome of the parent trial (low birth weight), we aim to enrol 12,712 participants 15–24 years per arm (25,424 total).

To monitor the micronutrient status of a subgroup of participants 15–18.9 years of age at enrolment, the sample size calculated for the reduction in anemia prevalence will be used (i.e., 1716 adolescents per arm). Stata software (StataCorp, College Station, TX, USA) has been used to randomly select 132 adolescents 15–18.9 years at enrolment from each cluster. Dietary intake will also be assessed in 200 randomly selected micronutrient status subgroup participants per arm (15 women per cluster), to further form a dietary status subgroup.

### Intervention and control

#### LSBE materials

In intervention clusters, enhanced LSBE materials have been developed on topics important to the empowerment of adolescent and young women to complement the existing LHW materials for use in community sessions. These will be implemented bi-monthly and attendance will be recorded. Three topic areas were prioritized: (a) delaying early marriage; (b) practicing appropriate personal and menstrual hygiene; and (c) the importance of good nutrition to health. Approximately 20 min is intended to be spent on each topic at community sessions. Integrated throughout the 3 topics are messages related to continuing one’s education, mental health (how to cope with stress, anxiety, and when you are upset), gender norms and equality, decision-making, advocacy, resiliency, participation, communication skills, facing challenges, agency, conflict resolution, and the prevention of violence.

Communication tools have been developed to assist the LHWs in conducting the LSBE-based community sessions. These were designed in coordination with Aahung, a Karachi-based non-governmental organization that aims to improve the sexual and reproductive health of girls (www.aahung.org; Karachi, Pakistan). Communication tools include a flipchart with 2 pictorials and discussion prompts per topic for use in sessions; and a brief summary pamphlet for distribution to participants. The flipcharts are similar to existing LHW tools for leading community sessions. A team of master trainers is trained on all LSBE materials and appropriate communication techniques. The master trainers provide comprehensive and interactive training for all intervention LHWs. Refresher training will also be provided on an ongoing basis throughout the duration of the trial.

#### Trial supplements and administration

The MMN supplements provided to participants in the intervention arm of the MaPPS Trial are consistent with the UNICEF/WHO/UNU international multiple micronutrient preparation (UNIMMAP), and have been procured from the UNICEF Supply Catalogue (https://supply.unicef.org). Each tablet is 10 mm in diameter and includes vitamin A: 800 μg RE; vitamin D: 5 μg; vitamin E: 10 mg; folic acid: 400 μg; vitamin B1 (thiamine): 1.4 mg; vitamin B2 (riboflavin): 1.4 mg; vitamin B3 (niacin): 18 mg; vitamin B12: 2.6 μg; vitamin B6: 1.9 mg; vitamin C: 70 mg; iron: 30 mg; zinc: 15 mg; iodine: 150 μg; copper: 2000 μg; selenium 65 μg (Micronutrient Tabs, Pregnancy; Lomapharm, Emmerthal, Germany). All MMN supplements are maintained and stored by the study manager in a locked study office that is temperature controlled. MMN supplements are provided to LHWs monthly by a study monitoring team, which is unblinded to the allocation of the intervention and not involved in data collection. Participants’ personal supply of MMN supplements are monitored and replenished every month by their respective LHWs. Once provided to participants, MMN supplements are stored in marked, opaque containers labelled with the participant’s ID and supplement batch number and expiry date.

Upon enrolment in the trial, intervention arm LHWs will visit new participants within 1 month to provide MMN supplements. Participants are asked to consume 1 MMN tablet 2 days/week. To make the incorporation MMN supplementation into their routine easier, participants are requested to choose a consistent time on 2 days in the week that are separate from each other (e.g., Monday and Thursday, or Wednesday and Saturday) on which to take 1 MMN tablet with a glass of water and a meal. Each participant is provided with a 1 month supply of MMN supplements, plus a spare week in case of unscheduled missed visits. As such, participants receive 10 MMN tablets per month (4 weeks/month × 2 tablets/week + 2 extra tablets = 10 tablets total). If a participant does not become pregnant within the context of the trial, she will take the MMN supplements for up to 24 months (the maximum duration of the preconception period).

Participants are instructed that the MMN supplements are just for them and should be kept away from children. They are asked to ensure that the lid of the supplement bottle is tightly closed when not in use and stored away from light and humidity. It is also explained that some people experience mild side effects when they start taking the MMN supplements (e.g., nausea, vomiting, diarrhea, stomach pains), but that taking it with food should make side effects less likely to happen. If they miss a dose, participants are instructed to take 1 MMN tablet the next time that they remember, but never to consume more than 1 tablet/day. If a participant has any side effects, is concerned, or has questions, she is instructed to contact her LHW and not study personnel since study personnel are blinded to whether participants are in the control or intervention arm. Participants are also asked not to take non-trial administered supplements while they are enrolled in the trial.

### Enrolment and consent

Adolescent and young women in LHW-covered areas in Matiari district were identified in a household listing exercise. Provisionally eligible participants will be invited to participate in MaPPS Trial with their family members and/or husbands present. Data collectors will explain the purpose and voluntary nature of the trial; participation components; and potential benefits and harms prior to obtaining written informed consent. If participants are < 16 years of age, assent will also be obtained as necessary. It is anticipated that enrolment will take at least 1 year.

### Visit schedule

All visits with study participants will occur at their homes (Table 1). Upon enrolment in the trial, a questionnaire designed to collect information on demographics; socioeconomic status (SES); reproductive health and history; life skills, empowerment, and social determinants of health (SDH)-related factors (Table 2); supplementation practices; and access to LHWs will be administered. This questionnaire has been adapted from the existing demographic health survey for Pakistan and several standardized assessment tools. All participants will also undergo anthropometric (height, weight, MUAC) and hemoglobin concentration measurement. After 12 months of enrolment in the preconception phase, all participants’ anthropometric measures (height, weight, MUAC) will be reassessed. After 24 months, all participants will be asked to complete a modified version of the baseline questionnaire for ongoing assessment of life skills, empowerment, and SDH-related factors; and undergo repeat anthropometric (height, weight, MUAC) and hemoglobin concentration measurements. Upon trial completion or withdrawal, participants will be asked to complete an exit questionnaire designed to assess their perception of their own health and satisfaction with trial participation.

**Table 1:** Preconception phase data collection and measurement activities

Activity	Month of participation			
	0	6	12	24
Consent	X			
Demographics and SES	X		M	X
Reproductive health and history	X			
Life skills, empowerment, and SDH	X		M	X
Anthropometry	X	M	X	X
Hemoglobin	X		M	X
Serum ferritin	M		M	M
Hepcidin	M		M	M
Transferrin receptor	M		M	M
Infection indicators (CRP, AGP)	M		M	M
Vitamin A	M		M	M
Vitamin D	M		M	M
Household food insecurity	X		M	X
Food group consumption survey (1 week)	D			
24-h recall	D			

X: all participants; *M* micronutrient status subgroup participants only; *D* dietary status subgroup participants only

**Table 2:** Factors relating to life skills, empowerment, and SDH captured within trial questionnaires

Domains	Includes information pertaining to	Evaluation tool
Nutrition	Household food insecurity	Household Food Insecurity Access Scale [17]
Health	Eating habits	Health Behaviour in School-aged Children [18]
	Body image	
	Physical activity	
Perception of support	Family, peer, and school	Multidimensional Scale of Perceived Social Support [19]
Decision making	Family, food, healthcare, and daily needs	Pakistan Demographic Health Survey [20]
Psychosocial health	Self-efficacy	Generalized self-efficacy scale [21]
	Stress	Perceived stress scale [22]
	Mental health (depression, anxiety, and stress)	Depression, Anxiety, and Stress Scale-21 [23]
Violence	Intimate partner violence	Conflict Tactics Scale [24]
	Exposure to violence	Pakistan Demographic Health Survey [20]

Additional measures will be collected from participants in the micronutrient status subgroup, including a 5 mL blood draw at enrolment and after 24 months; repeat anthropometric assessment (height, weight, MUAC) after 6 months; and a modified version of the baseline questionnaire for ongoing assessment of life skills and empowerment at 12 months. Participants in the dietary status subgroup will complete 3 dietary assessments (24-h food recall, weekly food group consumption survey) over the 3 weeks following enrolment in the trial. Dietary assessment days will be non-consecutive and unannounced, and include 2 weekdays and 1 weekend day.

#### LHW visits

Because LHWs are required to visit households in their catchment areas monthly, these visits will serve to monitor menses, new marriages, and suspected pregnancies throughout the duration of the trial, in addition to their mandated function [15]. All trial-administered supplement provision and consumption will also be recorded at these visits.

#### Adherence, morbidity, and morbidity monitoring

There will also be ongoing participant monitoring for the duration of the trial (Table 3). All participants will be visited by an independent surveillance team to collect morbidity and self-reported adherence data every 3 months. These visits will also allow for the observance and collection of data on non-trial administered nutritional supplement consumption.

**Table 3:** Monitoring data collection activities ongoing throughout the trial

Measurement	Monthly (LHW)	Quarterly (Monitoring Team)
Adherence by pill count^a^	i	
Adherence by self-reported frequency^b^		i
Side effects		X
Supplement acceptability		i
Morbidity		X
Mortality		X

X: all participants; i: intervention group only

^a^Adherence by pill count defined as number of tablets apparently consumed/number of possible supplements consumed given the number of days enrolled in the study

^b^Adherence options by self-report during preconception include twice weekly, intermittently, or not at all

### Data collection

Data collection tools have been customized for each type of visit, and data is collected orally given the low literacy rates in the trial population. Trained study personnel will collect all trial outcome data using questionnaires, anthropometric measurements, point-of-care tests, and dietary recall methods. Standardized operating procedures have been developed for all measures in an effort to make data collection practices consistent. Extensive training sessions will be provided to study personnel, employing classroom-based lectures, videos, hands-on practice, and mock interviews.

#### Questionnaires

For data collector-conducted visits with participants, tablets (Samsung Galaxy Tab. A T285; Samsung, Vietnam) will be used to collect questionnaire data. The tablets run a custom-made data collection application, which includes built-in logic and range checks. LHW monitoring visits will use paper-based questionnaires given the large number of LHWs involved in the trial. Dietary assessments will also be paper-based.

#### Anthropometric measurements

To assess adolescent and young women’s anthropometrics, height will be measured using a stadiometer (seca 213; seca, Hamburg, Germany); weight using a digital floor scale (seca 813); and MUAC using a measuring tape (seca 201). All measurements will be conducted in duplicate by 2 data collectors. In the case that 2 measurements exceed the pre-set allowable differences (height: < 1.0 cm; weight: < 0.5 kg; MUAC: < 0.5 cm), a third measurement will be obtained. The average (mean) of acceptable paired measures will be used in analysis.

#### Point-of-care hemoglobin assessment

To assess hemoglobin concentration, the HemoCue® Hb 301 System (HemoCue; Ängelholm, Sweden) will be used from blood collected via finger prick.

#### 24-h dietary recall

Among participants enrolled in the dietary assessment subgroup, usual dietary intake will be assessed using an interactive 24-h recall method [25] administered by trained food recall data collectors. Consumed food portions will be estimated and identified using a locally developed food kit and food item reference manual [26].

### Blood specimen collection and laboratory analyses

Trained phlebotomists (blinded to cluster allocation) will conduct all blood specimen collection using standard sampling procedures developed by the AKU Nutrition Research Lab. At each sampling time point, 5 mL of venous blood will be collected and stored in trace element-free vacutainer tubes (royal blue top; Greiner Bio-One, Monroe, NC, USA). Two drops of whole blood are immediately taken from the collection tube to assess hemoglobin concentration using the HemoCue® Hb 301 System point of care test. Samples will be further assessed for markers of nutritional status (iron [ferritin, transferrin receptor, hepcidin], vitamin A [retinol], and vitamin D [25(OH)D]) and inflammation (c-reactive protein, alpha-1-acid glycoprotein). More details on blood specimen collection and assays can be found within the description of the parent trial (Baxter et al., unpublished).

### Data management

Data collection tablets are collected from study personnel on a daily basis so that questionnaire data can be uploaded to the AKU data management unit. Paper-based questionnaire forms are visually checked by field site supervisors for completeness before being sent to the AKU data management unit on a weekly basis for entry into a database. Double data entry is used to reduce data entry errors. All collected data is kept under lock and key, and anonymized through the use of nine-digit participant identification codes. Data entered into the database is password protected.

### Outcome measures

To assess the prevalence of anemia, the primary outcome measures is hemoglobin concentration < 12 g/dL. There are many additional secondary outcomes within the trial related to health, nutrition, and empowerment that will be also assessed (see Additional file 1).

### Statistical analysis

For the primary outcome, the prevalence of anemia will be compared by intervention arm, irrespective of preconception supplementation duration or adherence (intention-to-treat). The per-protocol analysis will consider those women who complied with the recommend MMN supplementation frequency. Because the prevalence of anemia is high in the trial population, an individual-level analysis using generalized estimating equations (GEE) will be employed to determine the estimate of the population average. Clustering will be accounted for within the GEE analysis. There are several potential determinants which will be considered as covariates, such as age, parity, adherence, and SES. Relevant information will be collected at enrolment and throughout follow-up. Summary estimates (e.g., means, proportions, counts) will be reported with 95% confidence intervals. Dichotomous outcomes will be compared using risk ratios with 95% confidence intervals, and the difference in continuous variables will be determined by comparing means. *P* values less than 0.05 will be considered statistically significant. Statistical analyses will be conducted using Stata software. The plan for the analysis of secondary objectives and outcome measures will be presented elsewhere.

## Discussion

In this trial, we aim to assess the effectiveness of providing LSBE and MMN supplementation to non-pregnant adolescent and young women in rural Pakistan from preconception, and compare this intervention with the standard of care on anemia prevalence and other secondary health outcomes. The intervention will be implemented within the existing public health system, which is anticipated to lend to the interpretability of the trial findings and offer important insight around how to achieve coverage within the existing public health program context. This trial is distinctive in that it recruits both married and unmarried adolescent and young women, regardless of their intent to become pregnant, and collects measures on several domains of health and nutrition throughout participation.

The existing WHO guidelines on iron supplementation for non-pregnant adolescent and menstruating women can be confusing, particularly with respect to dosing frequency (weekly versus daily) and duration [27, 28]. At this time, we know of no guidelines that address preconception MMN supplementation. For adolescents, in particular, preconception care that includes MMN supplementation could be of benefit given that they are still developing and anemia can be caused by multiple different micronutrient deficiencies beyond iron [29, 30]. Including a culturally-tailored educational piece aimed at empowering the ability of participants to make informed decisions about their health, well-being, and nutrition will be key to sustained behavioural change throughout the life-course. Similarly, guidelines on pre-pregnancy nutritional counselling specific to adolescents are not known to exist, even though attending to adolescents’ nutritional needs will be important in addressing intergenerational malnutrition, chronic disease, and poverty [31]. Appropriate preconception care could allow that adolescents enter reproductive adulthood with improved health and nutrition status prior to conception.

Overall, the MaPPS Trial is expected to offer insight into providing an intervention that includes both LSBE and MMN supplementation to adolescent and young women from preconception and for a prolonged duration of time. We anticipate that there would be future follow up of the adolescent and young women in this cohort, so as to understand their long-term reproductive and empowerment outcomes, as well as development and disease outcomes. We also intend to investigate the cost-effectiveness of the provision of the described intervention.

### Trial status

As of May 2018, participants are still being enrolled to the trial.

## Additional file

Additional file 1 Additional tables detailing secondary outcome measures and cut-points. The additional file includes 3 tables detailing secondary nutritional status, anthropometric, and empowerment outcome measures for participants. (DOCX 23 kb)

## Abbreviations

AKU: Aga Khan University
GEE: Generalized estimating equations
LHW: Lady health worker
MaPPS Trial: Matiari emPowerment and preconception supplementation trial
MMN: Multiple micronutrient
MUAC: Middle-upper arm circumference
SES: Socioeconomic status
UNIMMAP: UNICEF/ WHO/UNU international multiple micronutrient preparation
WHO: World Health Organization
