# Supplementary material for: Feasibility and effect of life skills building education and multiple micronutrient supplements versus the standard of care on anemia among non-pregnant adolescent and young Pakistani women (15–24 years): a prospective, population-based cluster-randomized trial
Source: Reprod Health. 2018 May 30;15:103. doi: 10.1186/s12978-018-0547-y (PMC5977744; doi:10.1186/s12978-018-0547-y)
Supplement: Supplementary file 1 — Additional tables detailing secondary outcome measures and cut-points. The additional file includes 3 tables detailing secondary nutritional status, anthropometric, and empowerment outcome measures for participants. (DOCX 23 kb) [file 12978_2018_547_MOESM1_ESM.docx]

**Additional File 1 – MaPPS Anemia Protocol Manuscript**

**Table S1** Summary of nutritional status-related secondary outcome measures among subgroup participants

| Outcome | Type of variable | Variable definition | Analysis metric | Method of aggregation |
| --- | --- | --- | --- | --- |
| Iron status | Efficacy | Serum ferritin concentration <15 µg/L | Value at specified intervals | 1) Mean  2) % above or below reference range |
|  | Efficacy | Serum transferrin receptor concentration >4.4 mg/L | Value at specified intervals | 1) Mean  2) % above or below reference range |
|  | Efficacy | Hepcidin concentrations <2.0 ng/mL | Value at specified intervals | 1) Mean  2) % above or below reference range |
| Vitamin A status | Efficacy | Serum retinol concentration <0.70 μmol/l (VAD) or <0.35 μmol/l (severe VAD) | Value at specified intervals | 1) Mean  2) % above or below reference range |
| Vitamin D status | Efficacy | Serum 25(OH)D concentrations <50 nmol/L | Value at specified intervals | 1) Mean  2) % above or below reference ranges |
| Inflammation | Safety | Alpha-1-glycolytic protein concentrations >1.0 g/L | Value at specified intervals | 1) Mean  2) % above or below reference range |
|  | Safety | C-reactive protein concentrations >5.0 mg/L | Value at specified intervals | 1) Mean  2) % above or below reference range |
| Micronutrient intake^1^ | Efficacy | Intake of multiple micronutrients among women at set time points | Value at specified intervals | 1) Mean  2) % above or below reference ranges (EAR, RDA, UL) |

^1^Only determined among those participants in the dietary status subgroup

**Additional Table 2** Summary of anthropometric-related secondary outcome measures

| Outcome | Type of variable | Variable definition | Analysis metric | Method of aggregation |
| --- | --- | --- | --- | --- |
| Height | Efficacy | Height at set points in time | Value at specified intervals | 1) Mean  2) % below -2 SD^1^ (from z-score) |
| Weight | Efficacy | Weight at set points in time | Value at specified intervals | 1) Mean |
| MUAC | Efficacy | MUAC at set points in time | Value at specified intervals | 1) Mean |
| BMI | Efficacy | Derived from height and weight measurements at set points in time | Value at specified intervals | 1) Mean  2) % below -2 SD^1^ (from z-score) |

^1^Only determined among adolescent participants (age <19 years) using the WHO growth standards

**Additional Table 3** Summary of life skills-related and empowerment-related secondary outcome measures

| Outcome | Type of variable | Variable definition | Analysis metric | Method of aggregation |
| --- | --- | --- | --- | --- |
| Age at marriage | Empowerment | Age at which participant was first married | Value at specified intervals | 1) % married before 18 years of age  2) Mean |
| Completion of grade 10 schooling | Empowerment | Whether the participant has completed the 10^th^ grade | Value at specified intervals | 1) % completed 10^th^ grade  2) Mean grade completion |
| Use of sanitary pads | Empowerment | Use of a sanitary pad in the last two months during one’s menstrual cycle | Value at specified intervals | 1) % report using sanitary pad |
